# MACML: Marrying attention and convolution-based meta-learning method for few-shot IoT intrusion detection

**DOI:** 10.1371/journal.pone.0331065

**Published:** 2025-08-29

**Authors:** Congyuan Xu, Jun Yang, Panpan Li

**Affiliations:** 1 College of Artificial Intelligence, Jiaxing University, Jiaxing, Zhejiang, China; 2 School of Electrical and Information Engineering, Tianjin University, Tianjin, China; Firat University, TÜRKIYE

## Abstract

The widespread deployment of Internet of Things (IoT) devices has made them prime targets for cyberattacks. Existing intrusion detection systems (IDSs) heavily rely on large-scale labeled datasets, which limits their effectiveness in detecting novel attacks under few-shot scenarios. To address this challenge, we propose a meta-learning-based intrusion detection method called MACML (Marrying Attention and Convolution-based Meta-Learning). It integrates a self-attention mechanism to capture global dependencies and a convolutional neural network to extract local features, thereby enhancing the model’s overall perception of traffic characteristics. MACML adopts an optimization-based meta-learning framework that enables rapid adaptation to new tasks using only a small number of training samples, improving detection performance and generalization capability. We evaluate MACML on the CICIDS2018 and CICIoT2023 datasets. Experimental results show that, with only 10 training samples, MACML achieves an average accuracy of 98.75% and a detection rate of 99.17% on the CICIDS2018 dataset. On the CICIoT2023 dataset, it reaches 94.47% accuracy and a 95.32% detection rate, outperforming existing state-of-the-art methods.

## 1 Introduction

With the rapid development of the Internet of Things (IoT), its applications have expanded across various domains, including smart cities, intelligent manufacturing, smart homes, and intelligent transportation. However, the widespread deployment and openness of IoT devices also make them prime targets for cyberattacks. In recent years, IoT attacks have become increasingly diverse, including distributed denial of service (DDoS) attacks, malware propagation, data theft, and botnet intrusions, all of which pose significant threats to the security and stability of IoT systems [1]. Consequently, building an efficient and intelligent IoT intrusion detection system (IoT-IDS) has become a critical research topic in the field of network security.

Currently, IoT intrusion detection methods can be broadly classified into rule-based and machine learning-based approaches. Traditional rule-based methods rely on predefined attack patterns and detect intrusions through signature matching or anomaly behavior analysis. However, these methods struggle to handle novel attack patterns and often suffer from high false positive and false negative rates. In contrast, machine learning (ML)-based intrusion detection methods, especially those utilizing deep learning (DL) techniques, can automatically learn data features and exhibit better adaptability in complex network environments [2]. However, such methods often require large volumes of high-quality labeled data to achieve optimal performance. In real-world IoT environments, attack behaviors are highly dynamic and unpredictable, making it difficult to obtain sufficient labeled data for new attacks. As a result, DL-based models often face generalization issues when encountering previously unseen attack types. Additionally, deep learning models typically demand high computational resources, which limits their deployment on resource-constrained IoT devices [3]. Therefore, enhancing the detection and generalization capabilities of IDSs in few-shot learning scenarios remains a major challenge.

Recently, few-shot learning (FSL) has emerged as a promising solution to reduce the dependency of deep learning models on large-scale datasets. FSL enables models to quickly adapt to new tasks with limited labeled samples. Among various FSL approaches, meta-learning has gained significant attention in the field of IoT intrusion detection. The core idea of meta-learning is to “learn to learn” by training models on multiple tasks, enabling them to quickly adapt and generalize to new tasks with minimal data [4]. Several studies have explored the use of meta-learning for intrusion detection. Xu *et al.* proposed a metric-based first-order meta-learning framework, allowing IDS models to train across multiple tasks and improve their ability to detect novel attacks [5]. Sun *et al.* designed a prototype capsule network with a self-attention mechanism, integrating spatiotemporal feature fusion and prototype-based classification to enhance few-shot intrusion detection [6]. These studies demonstrate the potential of few-shot learning techniques in network intrusion detection. However, existing methods still face challenges such as limited feature extraction capability, insufficient generalization, and high computational complexity.

To address these challenges, we propose MACML (Marrying Attention and Convolution-based Meta-Learning), a novel few-shot IoT intrusion detection method. MACML integrates self-attention mechanisms and convolutional neural networks (CNNs) to leverage their respective advantages: the self-attention mechanism captures global dependencies between network traffic, while CNNs extract local features to compensate for the lack of local sensitivity in self-attention. Additionally, MACML employs an optimization-based meta-learning framework, where the model learns prior knowledge from known tasks during meta-training and quickly adapts to unknown tasks through fine-tuning, ensuring strong detection performance even in few-shot scenarios.

The main contributions of this paper are summarized as follows:

1. A novel intrusion detection method integrating self-attention and CNN. The proposed model combines a self-attention mechanism to capture global dependencies and a CNN to extract local features, enhancing the representation capability for complex network traffic.
2. An optimization-based meta-learning framework. The model learns prior knowledge from multiple training tasks and quickly adapts to new tasks, improving detection performance under few-shot conditions.
3. Implementation and evaluation of MACML-IDS. The proposed intrusion detection system, MACML-IDS, is validated on the CICIDS2018 and CICIoT2023 datasets. Experimental results show that MACML achieves high detection performance in few-shot scenarios and outperforms existing intrusion detection methods.

The remainder of this paper is organized as follows: Sect 2 reviews related work. Sect 3 describes the architecture, core components, and training process of MACML. Sect 4 presents the experimental setup and performance evaluation. Sect 5 discusses the research findings, and Sect 6 concludes the paper.

## 2 Related work

### 2.1 Meta-learning

Traditional deep learning models are typically trained from scratch using a fixed learning algorithm for a specific task. While this approach has achieved remarkable success in various domains, it presents significant limitations when dealing with data-scarce or high-cost annotation scenarios. Meta-learning, often referred to as “learning to learn” [4], aims to address this challenge by enabling models to generalize across multiple tasks, leveraging past experience to quickly adapt to new tasks with minimal data. In some sense, meta-learning is inspired by human cognitive processes, where individuals utilize prior knowledge to efficiently learn and adapt to novel situations. Unlike conventional deep learning, which heavily relies on large-scale labeled data for each task, meta-learning focuses on rapid adaptation, improved generalization, and efficient transfer of learned knowledge to new problems.

Meta-learning methods can generally be classified into optimization-based, model-based, and metric-based approaches [7].

Optimization-based meta-learning methods treat the learning process as a bi-level optimization problem, where a meta-learner optimizes the model parameters to enable fast adaptation to new tasks with minimal updates. Representative approaches include MAML [8], MAML++ [9], FOMAML [10], L2O [11], and BOIL [12]. MAML, for example, learns an effective initialization of model parameters such that only a few gradient updates are needed to adapt to a new task. However, despite its flexibility, MAML suffers from high computational costs due to the need for second-order derivatives. FOMAML improves upon MAML by approximating first-order gradients, reducing computational overhead while potentially introducing performance trade-offs. L2O (Learning to Optimize) utilizes recurrent neural networks to learn task-specific optimization rules, allowing for adaptive gradient updates and improved learning efficiency.

Model-based meta-learning, on the other hand, utilizes neural network architectures that explicitly capture task relationships and dynamically update their internal states to facilitate fast adaptation. These models maintain an internal memory representation of prior tasks, which is updated when encountering new data. Due to their reliance on learned task embeddings, these methods are often referred to as “black-box models.” Representative approaches include MANNs [13] and SNAIL [14]. MANNs (Memory-Augmented Neural Networks) combine deep learning with external memory components to store and retrieve task information efficiently. SNAIL (Simple Neural Attentive Meta-Learner) integrates attention mechanisms with convolutional layers to improve meta-learning performance. While these methods are effective for a variety of tasks, they often lack interpretability due to their complex internal representations.

Metric-based meta-learning focuses on learning a feature space where task adaptation is performed using similarity measures rather than directly updating model parameters. These methods rely on computing similarity scores between unseen samples and known samples in the feature space. Representative works include Siamese Networks [15], Triplet Networks [16], Prototypical Networks [17], RelationNet [18], ATL-Net [19], and DeepBDC [20]. Siamese Networks leverage twin neural networks to measure pairwise similarity, while Triplet Networks improve upon this by considering relative distances among multiple instances. Prototypical Networks learn class prototypes in an embedding space, enabling efficient classification of new samples based on their proximity to known prototypes. Since metric-based approaches do not require explicit parameter updates, they are computationally efficient and well suited for real-time applications.

Compared to traditional deep learning, meta-learning offers a more flexible approach to model generalization, making it highly suitable for IoT intrusion detection in few-shot scenarios. Table 1 summarizes representative meta-learning related works discussed in this study.

**Table 1:** Summary of meta-learning related works.

Study	Method	Key contribution
Yao *et al.* [7]	Meta-learning taxonomy	Categorized meta-learning into optimization, model, and metric-based approaches
Finn *et al.* [8]	MAML	Introduced model-agnostic meta-learning enabling fast adaptation with few gradient updates
Antoniou *et al.* [9]	MAML++	Improved MAML with stable training strategies and performance gains
Nichol [10]	FOMAML	Simplified MAML with first-order gradient approximation for lower cost
Chen *et al.* [11]	L2O	Used RNN-based optimizer to learn adaptive gradient update rules
Oh *et al.* [12]	BOIL	Proposed feature reuse and decoupled reinitialization to boost generalization
Santoro *et al.* [13]	MANNs	Combined neural nets with external memory to enable rapid task adaptation
Mishra *et al.* [14]	SNAIL	Integrated attention and temporal convolutions for flexible meta-learning
Koch *et al.* [15]	Siamese Network	Used twin networks to compute similarity for few-shot classification
Hoffer and Ailon [16]	Triplet Network	Trained embedding via relative distances between anchor, positive, negative samples
Snell *et al.* [17]	Prototypical Network	Represented each class by its prototype for fast inference
Sung *et al.* [18]	RelationNet	Learned a deep distance metric between support and query samples
Dong and Yang [19]	ATL-Net	Used attentional transformation to improve few-shot classification
Xie *et al.* [20]	DeepBDC	Exploited second-order statistics in embedding space to enhance discriminability

### 2.2 Self-attention mechanism

The attention mechanism, inspired by human cognitive processes, selectively focuses on important aspects of input data while suppressing less relevant information. This concept has been widely adopted in various fields, including machine translation, image processing, and speech recognition. The core principle of attention mechanisms is to compute interdependencies among input elements and assign different weights to them, allowing models to prioritize key information and improve performance.

Self-attention mechanisms, particularly those introduced in the Transformer model [21], have demonstrated remarkable success in capturing long-range dependencies within sequential data. The self-attention mechanism encodes input sequences into query (*Q*), key (*K*), and value (*V*) representations, computes similarity scores between queries and keys, and generates weighted representations based on these scores. This enables the model to learn relationships between distant elements in the input data, resulting in more expressive feature representations. Self-attention mechanisms have been extensively utilized in deep learning architectures such as BERT [22] and the GPT series [23–26] for natural language processing, as well as ViT [27], SENet [28], and DenseNet [29] for image recognition. Continued advancements in attention-based architectures, particularly the GPT series, have significantly contributed to the development of large-scale pre-trained models.

Despite their strong performance, self-attention models still face several challenges. First, they are often designed for specific tasks, which limits their generalization capabilities, particularly in low-level vision tasks. Second, the quality of training data has a direct impact on model effectiveness, and poor-quality data can degrade generalization performance. Additionally, self-attention mechanisms involve high computational costs, making them resource-intensive for large-scale data processing, which poses challenges for real-time applications. Lastly, integrating multi-modal data and handling multi-task learning effectively remain open research questions. Many existing self-attention models are optimized for single-task learning and struggle with fusing information across multiple modalities. Furthermore, for certain specific tasks, self-attention models may not outperform conventional convolutional neural networks of similar scale. A summary of self-attention related works is presented in Table 2.

**Table 2:** Summary of self-attention related works.

Study	Method	Key contribution
Vaswani *et al.* [21]	Transformer	Introduced self-attention for capturing long-range dependencies in sequences
Kenton and Toutanova [22]	BERT	Used bidirectional transformers for deep contextual language representation
Radford *et al.* [23]	GPT-2	Applied large-scale autoregressive transformers to language generation
Brown *et al.* [24]	GPT-3	Demonstrated few-shot learning via large-scale pre-trained transformers
Ouyang *et al.* [25]	InstructGPT	Aligned GPT with human preferences using reinforcement learning from feedback
Achiam *et al.* [26]	GPT-4	Improved reasoning and instruction-following with larger multimodal models
Dosovitskiy *et al.* [27]	ViT	Applied pure transformer architecture to image classification
Hu *et al.* [28]	SENet	Enhanced channel-wise feature recalibration through squeeze-and-excitation blocks
Huang *et al.* [29]	DenseNet	Improved feature reuse and gradient flow via dense connectivity

### 2.3 Few-shot IoT intrusion detection

IoT intrusion detection remains an emerging research area, and existing detection methods, whether traditional, machine learning-based, or deep learning-based, still have considerable room for improvement. Traditional intrusion detection techniques often suffer from high false positive and false negative rates, whereas machine learning and deep learning-based approaches require large labeled datasets, substantial computational resources, and extended training times. Moreover, these models are usually trained for specific attack types and struggle to detect unseen threats. Few-shot learning offers a potential solution to these challenges by reducing the dependency on large training datasets while maintaining detection accuracy.

Recently, researchers have begun to explore the integration of few-shot learning techniques into IoT intrusion detection. Xu *et al.* proposed FC-Net, which learns a pair of feature maps for classification from a pair of network traffic samples and determines whether the samples belong to the same type [30]. Zhang *et al.* proposed a malware traffic classification method combining knowledge transfer with neural architecture search, enabling adaptive feature extraction for new attack patterns [31]. Wang *et al.* introduced BT-TPF, a knowledge distillation-based IoT intrusion detection model that utilizes a Siamese network to reduce the dimensionality of complex, high-dimensional network traffic data [32]. It further employs a lightweight PoolFormer classifier under the guidance of a large-scale Vision Transformer to lower computational costs. Li *et al.* proposed a hybrid CNN-LSTM-GAN-based DoS intrusion detection system, combining signature-based and anomaly-based detection to improve detection performance for both known and unknown DoS attacks [33]. Shen *et al.* designed a DQN-based heuristic learning IDS (DQN-HIDS) for edge-based SIoT networks, which calculates sample similarity through SIoT processing modules and optimizes detection strategies using a deep Q-network (DQN) with LSTM [34]. Mao *et al.* proposed a label-aware federated graph contrastive learning framework named FeCoGraph that leverages line graphs, contrastive objectives, and federated learning to enable few-shot intrusion detection while preserving data privacy. [35]. Li *et al.* proposed FedGen+, an improved generative federated distillation framework for IoT intrusion detection that addresses data heterogeneity and privacy concerns by using a server-trained generator to augment client-side training without requiring a proxy dataset [36].

These studies demonstrate that combining multiple techniques often leads to superior performance compared to single-method approaches. Therefore, we build upon this idea by integrating convolutional neural networks and self-attention mechanisms with meta-learning to propose a novel IoT intrusion detection method that improves both detection accuracy and generalization capability. An overview of recent few-shot IoT intrusion detection works is provided in Table 3.

**Table 3:** Summary of few-shot IoT intrusion detection works.

Study	Method	Key contribution
Xu *et al.* [30]	FC-Net	Compared feature map pairs to detect whether two traffic samples belong to the same class using few-shot learning
Zhang *et al.* [31]	Enhanced FSL	Combined knowledge transfer and neural architecture search to classify malware traffic with limited data
Wang *et al.* [32]	BT-TPF	Used Siamese networks and PoolFormer under ViT guidance for lightweight few-shot intrusion detection
Li *et al.* [33]	HDA-IDS	Proposed hybrid CNN-LSTM-GAN model to detect both known and unknown DoS attacks
Shen *et al.* [34]	DQN-HIDS	Combined LSTM-based Q-learning and similarity computation for edge-based SIoT intrusion detection
Mao *et al.* [35]	FeCoGraph	Proposed a label-aware federated graph contrastive learning framework using line graphs and contrastive objectives to enable privacy-preserving few-shot intrusion detection
Li *et al.* [36]	FedGen+	Introduced a generative federated distillation method that augments local training with server-learned representations to handle heterogeneity

## 3 Method

### 3.1 Overview

We propose a few-shot IoT intrusion detection method named MACML (Marrying Attention and Convolution-based Meta-Learning), designed to address the challenges posed by limited training data in IoT security. MACML leverages the strengths of both the self-attention mechanism and convolutional neural networks to enhance detection performance and generalization capability in complex network traffic environments. Specifically, the self-attention mechanism is employed to capture long-range dependencies between network traffic features, while CNNs are used to extract local spatial patterns, compensating for the self-attention mechanism’s limitations in local feature extraction.

Additionally, MACML incorporates a data preprocessing module to clean, normalize, and transform raw network traffic data, ensuring high-quality inputs for the detection model. During training, MACML follows a meta-learning framework, in which the model learns prior knowledge from multiple training tasks and subsequently fine-tunes itself to adapt quickly to unseen tasks. This learning-to-learn approach enables the model to achieve strong detection performance even in low-data scenarios, reducing its reliance on large-scale labeled datasets.

**Fig 1:**
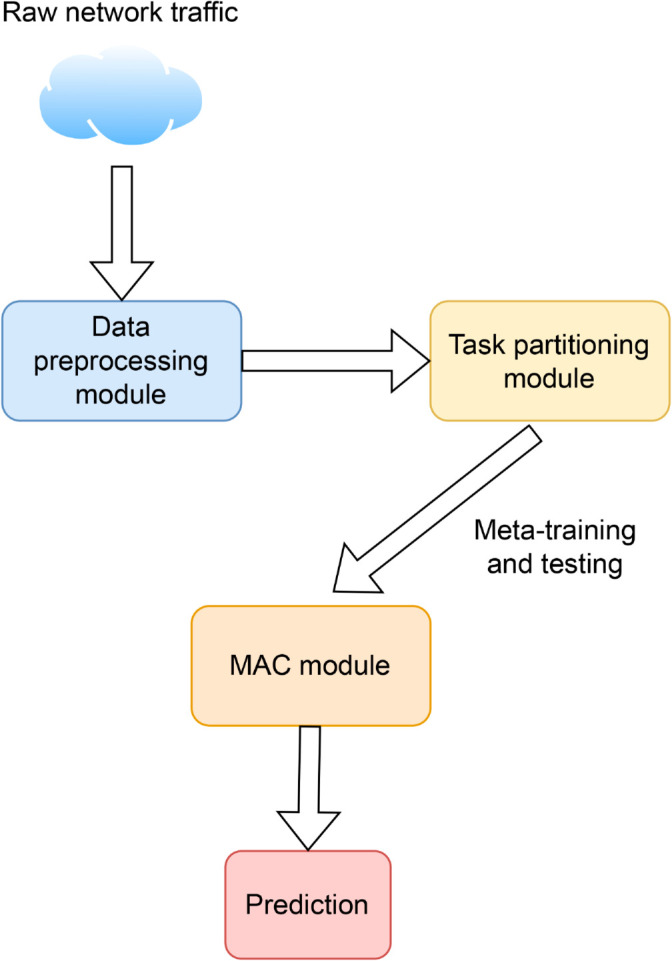
The overall architecture of MACML-IDS.

The overall architecture of MACML-IDS is illustrated in Fig 1, consisting of three main components: the preprocessing module, the task partitioning module, and the MAC module. The MAC module, which integrates self-attention and CNN, serves as the core component of the system and is responsible for extracting both global dependencies and local features to improve detection robustness. The following sections describe each module in detail.

### 3.2 Data preprocessing module

The input to MACML is raw network traffic data in PCAP format, which must undergo preprocessing before being fed into the neural network. The data preprocessing module follows these key steps:

1. **Data flow segmentation:** Raw PCAP files are segmented into individual data flows based on source IP, destination IP, protocol, source port, and destination port to ensure data integrity and consistency.
2. **Address anonymization:** IP addresses and port numbers are anonymized to eliminate sensitive information while preserving essential traffic characteristics. In this study, a zero-masking technique is applied to anonymize address-related features.
3. **Packet extraction and padding:** To standardize data input formats, each data flow extracts the first 16 packets, with each packet containing the first 256 bytes. If a packet is shorter than 256 bytes, zero-padding is applied to ensure a fixed-length representation.

Through these preprocessing steps, the final structured dataset ensures high data quality and consistency, providing reliable input for the MACML-IDS model across different IoT environments.

### 3.3 Task partitioning module

Since the proposed method is based on optimized meta-learning, the core idea is to train the model across multiple meta-tasks, enabling it to acquire task-agnostic knowledge that facilitates fast adaptation to new attack types. Before training, the dataset is partitioned into different tasks to construct meta-training and meta-testing sets.

The task partitioning process follows these steps:

1. **Dataset splitting:** The preprocessed dataset is divided into a training set and a testing set to ensure proper model evaluation.
2. **Training task construction:**
  - Randomly select two traffic classes (one attack type and one benign traffic type) from the training set.
  - Further split the selected data into a support set (few-shot training samples) and a query set (evaluation samples for meta-learning).
  - Repeat this process multiple times to generate diverse training tasks.
3. **Testing task construction:** The testing set is partitioned using the same method as the training set. However, unlike training tasks, the testing set contains attack types that were not seen during training, ensuring that the model’s generalization ability is properly evaluated.

By adopting this task-based partitioning strategy, the model can learn common attack patterns across different training tasks and effectively adapt to new attack types during evaluation, significantly improving its robustness and generalization capability.

### 3.4 MAC module

The MAC module is designed to fully exploit the advantages of self-attention mechanisms and CNNs, addressing their respective weaknesses while enhancing their strengths. The self-attention mechanism effectively captures global dependencies in network traffic data, while CNNs are employed to extract local spatial patterns, ensuring a comprehensive feature representation.

**Fig 2:**
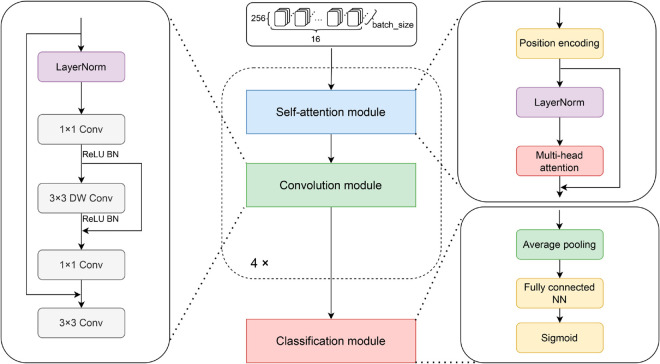
The overall architecture of the MAC module.

The structure of the MAC module is depicted in Fig 2. It consists of three major modules: (1) Self-attention module; (2) Convolution module; (3) Classification module.

The self-attention module includes position encoding, layer normalization, and multi-head attention mechanisms. Position encoding is applied to retain the sequential order of network packets, as shown in Eq 1.

$$PE(p{)}^{\left(i\right)}=\left\{ \begin{array}{cc} \sin\left(\frac{p}{1000^{\frac{2k}{d}}}\right), & i=2k+1\\ \cos\left(\frac{p}{1000^{\frac{2k}{d}}}\right), & i=2k \end{array}\right.$$ (1)

where *p* represents the *p*-th element in the input $x_{1}\ldots x_{16}$, *d* is the dimension of each flow, and *i* denotes the position of each byte in a single flow. The terms sin(·) and cos(·) ensure that the positional information is bounded within a limited range.

Following position encoding, layer normalization and residual connections are applied to the input flows $x_{1}^{\prime },\ldots ,x_{16}^{\prime }$ to accelerate information transmission and facilitate faster convergence. Subsequently, multi-head self-attention is performed. Multi-head self-attention computes multiple self-attention operations in parallel. The self-attention operation proceeds as follows: for each input network traffic sample, linear transformations are applied to obtain three vector matrices—Query, Key, and Value. First, the Query vector is multiplied by the Key vector, followed by layer normalization, to compute attention weights. These weights are then multiplied by the Value vector, and residual connections are applied to obtain the weighted value representation, thereby enhancing feature transfer capability. The self-attention mechanism effectively captures global dependencies within the raw traffic. The specific computation is given in Eq 2.

$$\text{Attention}(Q,K,V)=\text{softmax}\left(\frac{QK^{T}}{\sqrt{d_{k}}}\right)V$$ (2)

where *Q*, *K*, and *V* are the weight parameters generated through linear transformations, used to compute the relevance of each traffic flow.

Multi-head self-attention splits the input vectors into multiple subspaces, where attention is computed independently and in parallel. The input vectors $x_{1}^{\prime }\ldots x_{16}^{\prime }$ are divided into smaller-dimensional inputs $y_{1}^{1}\ldots y_{16}^{1}\ldots y_{1}^{h}\ldots y_{16}^{h}$, where *h* represents the number of attention heads. Each subspace is known as a head, and the resulting attention weights from each head are concatenated to form the final self-attention matrix, which is then multiplied by the weight matrix *W*_0_. This approach prevents the model from learning only partial information, as it captures various aspects of the data. The specific computation is expressed in Eq 3.

$$\text{MultiAttention}=W_{m}{\left[a_{1}\ldots a_{h}\right]}^{T}$$ (3)

where *a*_*i*_ represents the self-attention matrix of the *i*–*th* head, and *W*_*m*_ is the weight matrix generated by linear transformations for multi-head self-attention.

To accelerate model convergence and prevent issues such as vanishing gradients, residual connections are applied both before layer normalization and after multi-head self-attention operations.

While the self-attention mechanism captures global dependencies between flows, it overlooks local information. To address this limitation, we employ convolutional neural networks to extract local features. The convolution module, similar to the self-attention module, also incorporates layer normalization and residual connections. To reduce the number of parameters in the convolution module, a depthwise separable convolution-based residual feedforward neural network is used. This architecture not only extracts local features but also approximates the performance of traditional CNNs while reducing model parameters and training time.

The specific structure of the convolution module is shown in Fig 2. It begins with layer normalization, followed by two 1×1 convolutional kernels and a depthwise separable 3×3 convolution, which extracts local features. Residual connections are applied in the middle to speed up information transmission. Finally, a 3×3 convolution is stacked for downsampling, further extracting local features. The use of ReLU activation and batch normalization prevents overfitting. The computation is shown in Eq 4:

RFFN(x)=Conv(f(Conv(x)))f(x)=DWConv(x)+(x) (4)

Unlike CNNs used in image recognition, which reduce the image size while retaining important information, MACML processes network traffic, which is closer to natural language than images. Therefore, the convolution module does not reduce the scale of the transmitted information, ensuring that more useful information is retained for both the self-attention and convolution modules to extract global and local features, respectively.

The classification module reduces the dimensionality of the feature information extracted by the aforementioned two modules. Using average pooling and a fully connected neural network, the classifier extracts and reduces features from the convolutionally processed data, maintaining important information while reducing computational load. Finally, the Sigmoid function is applied for binary classification prediction.

### 3.5 MACML training process

**Fig 3:**
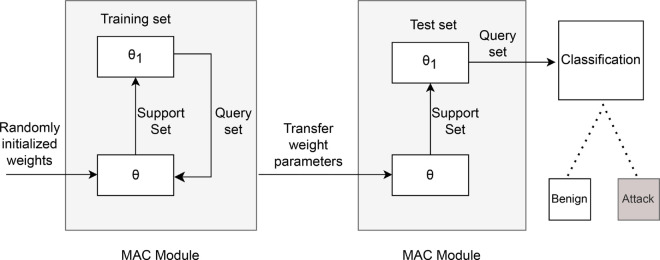
Training process of the MAC module.

The training process of MACML differs from that of traditional deep learning methods. The training procedure of the proposed method is illustrated in Fig 3. During the meta-training phase, the model’s initial weight parameters θ are randomly initialized. Then, the loss of the MAC module on the support set for known tasks is computed, and the weight parameters are updated using gradient descent and backpropagation algorithms to obtain the updated parameters $\theta_{1}$. At this point, the updated weight parameters $\theta_{1}$ are not directly used in the model. Instead, the loss is calculated on the query set to prevent overfitting on the support set. Subsequently, gradient descent and backpropagation are applied for a second round of updates. This second update directly alters the model’s weight parameters, transitioning from the initialized parameters θ to the trained initialization parameters. In this process, the weight parameters are updated based on the loss of each data sample in the support set, while for the query set, the loss across all data is used to update the parameters. The primary goal of this training process is to enable the model to find weight parameters θ that are relatively good across all tasks. This allows the model to quickly adapt to new tasks through fine-tuning, thereby demonstrating the ability to generalize effectively from one task to another.

After the model has been trained for a certain number of epochs on the training set, fine-tuning and testing are performed using the test set. The test set is partitioned in the same way as the training set and also includes both support and query sets. However, the data types in the test set are not present in the training set. During the meta-testing phase, the weight parameters θ obtained during meta-training are used as the initialization parameters for the test phase. The model is then fine-tuned on the support set using a small number of target task samples, resulting in updated weight parameters $\theta_{1}$. The model is evaluated on the query set to obtain the classification results. The primary goal during training is to obtain an initialized weight parameter that can be fine-tuned during meta-testing to generalize to the target task. The entire training process is summarized in Eq 5.

$$p(D_{T}^{q}|\theta ,D_{T}^{t})=\prod_{\tau \in T}p(D_{\tau }^{q}|\theta_{\tau }^{\prime }=\theta +\alpha \nabla_{\theta_{0}}\log p(D_{\tau }^{s}|\theta ))$$ (5)

where $p\left(D_{\tau }^{s}|\theta_{0}\right)$ represents the posterior knowledge learned by MACML on the support set. By continuously updating the parameters θ, MACML learns more posterior knowledge, which is then used as the prior knowledge for MACML on the query set $D_{T}^{q}$, allowing it to obtain the posterior knowledge on the query set $D_{T}^{q}$. $D_{T}^{t}$ represents the training set.

## 4 Evaluation

### 4.1 Dataset

To evaluate the effectiveness of the proposed MACML-IDS, we utilize two datasets: the CICIoT2023 dataset, which contains real-world IoT attack scenarios, and the widely used CICIDS2018 dataset from the network security field. The following provides a brief overview of the datasets used in this study.

The CICIoT2023 dataset was introduced by the Canadian Institute for Cybersecurity in 2023 to facilitate security analysis applications in real IoT environments [37]. The dataset consists of network traffic from 105 IoT devices, where 33 attacks were executed, categorized into seven types: DDoS, DoS, Recon, Web-based, Brute Force, Spoofing, and Mirai. Since some data labels may have quality issues, we selected representative attack types with high impact for our experiments. The data types are listed in Table 4.

**Table 4:** Data types in CICIoT2023 dataset.

Data type	Description
Mirai	Botnet attack
DoS	Denial-of-Service attack
Spoofing	Spoofing attack
Recon	Reconnaissance attack
Benign	Normal traffic

The CICIDS2018 dataset, a collaborative project between the Communication Security Establishment (CSE) and the Canadian Institute for Cybersecurity, was designed to overcome the limitations of anonymized datasets and improve intrusion detection system evaluation [38]. It includes seven types of attacks: Brute Force, Heartbleed, Botnet, DoS, DDoS, Web Attacks, and Infiltration. Similar to CICIoT2023, we selected high-impact attack types for our experiments, as shown in Table 5.

**Table 5:** Data types in CICIDS2018 dataset.

Data type	Description
DDoS	Distributed Denial-of-Service attack
DoS	Denial-of-Service attack
Brute Force	Password cracking attack
Botnet	Botnet attack
Benign	Normal traffic

### 4.2 Evaluation metrics

To assess the performance of the proposed intrusion detection model, we employ five commonly used evaluation metrics: Accuracy, Detection Rate, Precision, Specificity, and F1-Score. These metrics are defined as follows:

- **Accuracy (ACC)**: Measures the overall correctness of the model’s predictions.
  $$\text{Accuracy}=\frac{TP+TN}{TP+TN+FP+FN}$$ (6)
- **Detection Rate (DR)**: Also known as recall, this metric quantifies the model’s ability to correctly identify positive samples.
  $$\text{Detection Rate}=\frac{TP}{TP+FN}$$ (7)
- **Precision (PR)**: Evaluates the proportion of correctly identified positive samples among all samples predicted as positive.
  $$\text{Precision}=\frac{TP}{TP+FP}$$ (8)
- **Specificity (SPEC)**: Measures the model’s ability to correctly classify negative samples.
  $$\text{Specificity}=\frac{TN}{TN+FP}$$ (9)
- **F1-Score**: The harmonic mean of Precision and Detection Rate, providing a balanced measure of classification performance.
  $$\text{F1-Score}=\frac{2\;\times \;\text{Precision}\;\times \;\text{Detection Rate}}{\text{Precision}+\text{Detection Rate}}$$ (10)

Here, TP (True Positives) represents correctly identified attack samples, TN (True Negatives) denotes correctly identified benign samples, FP (False Positives) corresponds to benign samples incorrectly classified as attacks, and FN (False Negatives) represents attack samples incorrectly classified as benign. These metrics comprehensively evaluate the performance of MACML-IDS in detecting IoT-based network intrusions.

To demonstrate the effectiveness of our selected evaluation metrics and facilitate meaningful comparisons, we review representative works in recent literature. Most studies on IoT intrusion detection using few-shot or meta-learning approaches, such as FC-Net [30], BT-TPF [32], and HDA-IDS [33], primarily adopt metrics such as accuracy, precision, detection rate (recall), and F1-score. These metrics are essential for handling imbalanced traffic data and identifying both common and rare attack types.

Unlike traditional accuracy-focused evaluations, our study emphasizes a balanced assessment by jointly considering precision, detection rate, and F1-score, which better reflect the trade-off between false positives and false negatives. For example, BT-TPF [32] focuses on model compression and lightweight deployment, whereas other works such as HDA-IDS [33] and FC-Net [30] report strong detection performance but often omit specificity. In contrast, our evaluation includes specificity to better assess the model’s ability to distinguish normal traffic from malicious behavior, which is essential for real-world IoT environments.

This comprehensive metric selection not only aligns with widely accepted evaluation practices but also enables fair and interpretable comparisons with state-of-the-art methods in IoT intrusion detection.

### 4.3 Experimental setup

Our experiments were conducted on a system equipped with an AMD EPYC 9754 128-Core Processor, an RTX 4090D (24 GB) GPU, and 64 GB of RAM. We utilized CUDA 11.2 for GPU acceleration and PyTorch 2.1.2 for model training and inference. The system was operated on Ubuntu 20.04 with Python 3.8. The hyperparameters used for training our intrusion detection model are listed in Table 6.

**Table 6:** Hyperparameter settings for MACML-IDS.

Hyperparameter	Value
*α* (Inner learning rate)	$4\;\times \;10^{-2}$
*β* (Outer learning rate)	$2\;\times \;10^{-4}$
Epochs	100
Attention heads	8
Batch size	10

We conducted binary classification experiments on both CICIoT2023 and CICIDS2018 datasets. To comprehensively evaluate the intrusion detection system’s performance, we designed the following two types of experiments:

- **Experiment I**: Same-domain evaluation. Analysis of accuracy and detection rate trends during training, as well as final evaluation metrics on the test set.
- **Experiment II**: Cross-domain evaluation. Real-world scenario simulation by training on one dataset and testing on another.

### 4.4 Experimental results

#### 4.4.1 Results of experiment I.

**Fig 4:**
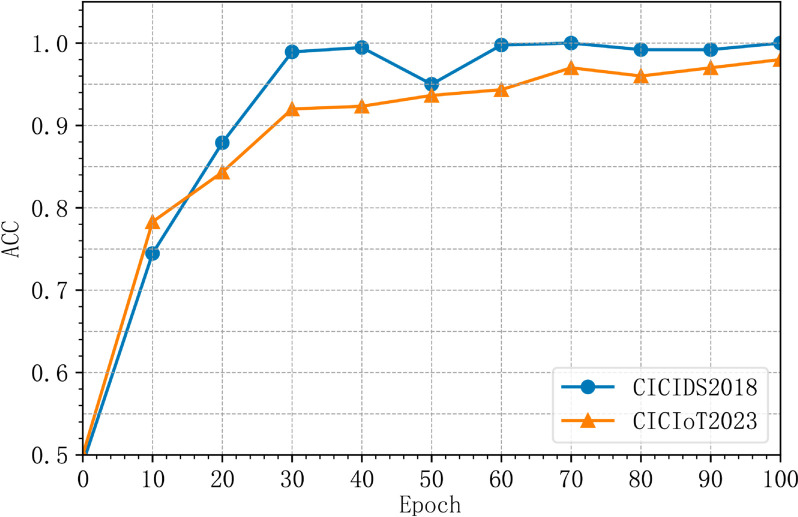
Accuracy trends during training.

**Fig 5:**
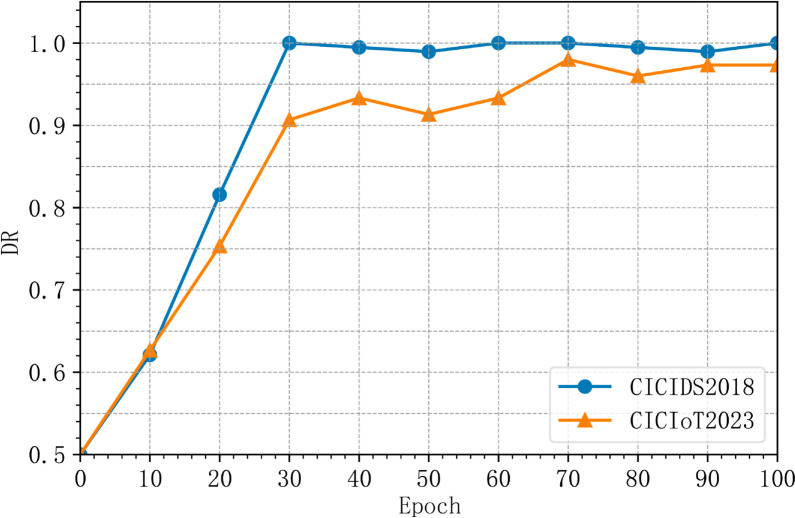
Detection rate trends during training.

In Experiment I, we analyzed the accuracy and detection rate trends during the training process. As shown in Figs 4 and 5, MACML-IDS achieves rapid convergence within the first 30 training epochs, after which the performance stabilizes with minimal fluctuations.

From Figs 4 and 5, it is evident that during the initial 0 to 30 training epochs, the model’s accuracy and detection rate increase rapidly. Between epochs 30 and 100, minor oscillations occur, but the overall trend remains positive. At 100 epochs, MACML-IDS reaches a stable state, demonstrating its ability to converge efficiently while maintaining high detection performance.

**Fig 6:**
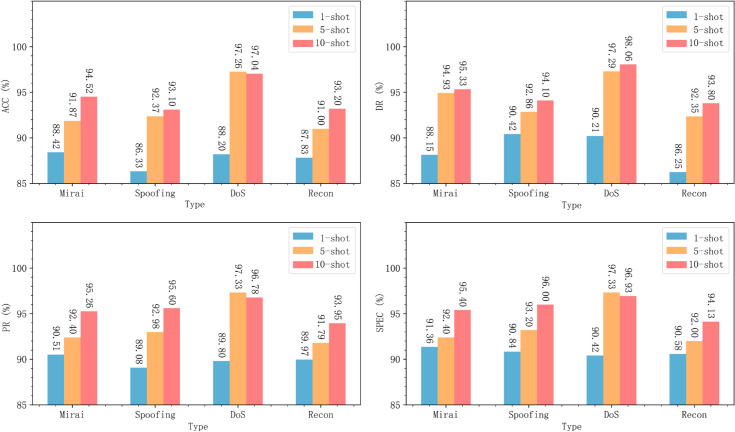
MACML-IDS performance evaluation on CICIoT2023 dataset with different training sample sizes.

To evaluate the detection performance after training, we tested MACML-IDS on the CICIoT2023 dataset using different numbers of training samples (1, 5, and 10). The accuracy, detection rate, precision, and specificity achieved in these cases are summarized in Fig 6.

To minimize errors, we conducted multiple test rounds and averaged the results. As shown in Fig 6, even with only one training sample, MACML-IDS achieves a detection rate as high as 88.76%. When the number of training samples increases to 5 and 10, the detection rate further improves, reaching a maximum of 96.34%. The specificity remains above 90% in all tested scenarios, demonstrating the robustness and reliability of our method.

**Fig 7:**
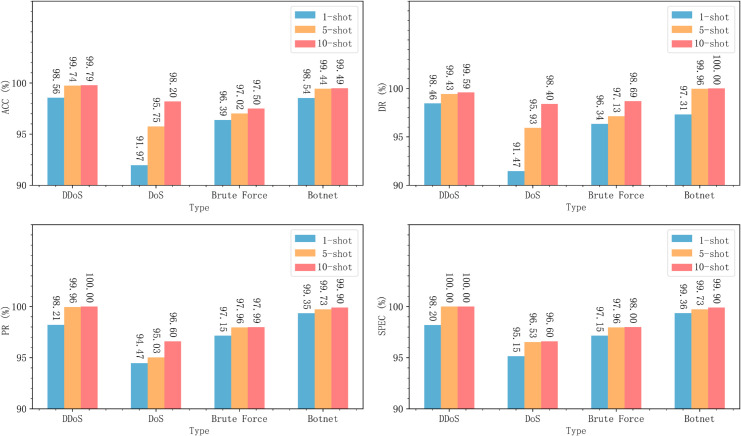
MACML-IDS performance evaluation on CICIDS2018 dataset with different training sample sizes.

Similar experiments were conducted on the CICIDS2018 dataset, and the results are shown in Fig 7.

For the CICIDS2018 dataset, even with only one training sample, MACML-IDS achieves an accuracy of up to 95.90%, and all other evaluation metrics exceed 95%. When the number of training samples increases to 5 and 10, the model’s accuracy reaches 97.99% and 98.75%, and the detection rate improves up to 99.17%. These results confirm that MACML-IDS performs effectively on both datasets.

Table 7 presents the F1-score of MACML-IDS for different attack types with different numbers of training sample sizes.

**Table 7:** F1-score for different attack types with different training sample sizes.

Data type	1-shot	5-shot	10-shot
DDoS	98.3	99.7	99.8
DoS (2018)	93.0	95.4	97.5
Brute Force	96.7	97.5	98.3
Botnet	98.3	99.8	99.9
Mirai	90.9	92.4	95.3
Spoofing	89.9	93.1	95.8
DoS (2023)	90.1	97.3	97.1
Recon	90.3	91.9	94.0

The lowest F1-score in the table exceeds 89.9%, and with 10 training samples, MACML-IDS achieves an F1-score of up to 99.9%, indicating its ability to effectively distinguish between attack and benign traffic with a low false positive rate.

**Fig 8:**
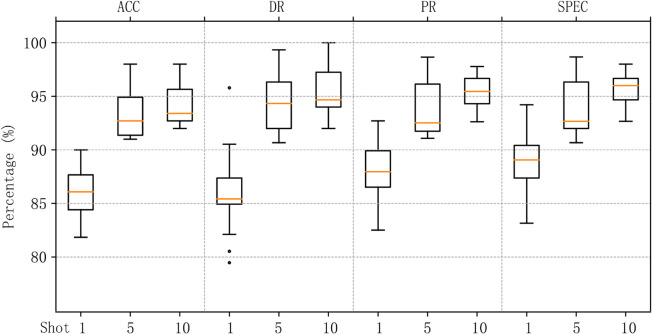
Data distribution of the results of testing all attack types (CICIoT2023).

**Fig 9:**
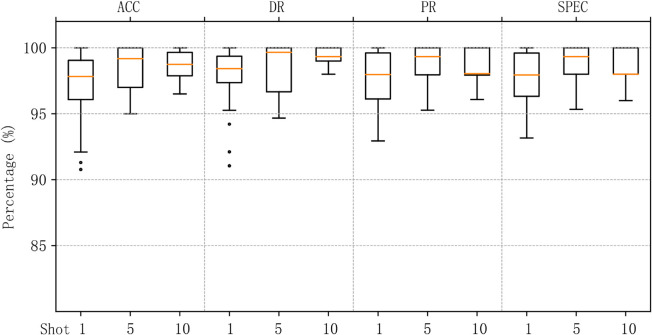
Data distribution of the results of testing all attack types (CICIDS2018).

We aggregated the results of MACML-IDS tested on different attack types and analyzed the data. Fig 8 represents the data distribution on the CICIoT2023 dataset, and Fig 9 represents the data distribution on the CICIDS2018 dataset. In the 1-shot scenario, outliers were observed in both the CICIoT2023 and CICIDS2018 datasets in terms of accuracy and detection rates. However, no outliers appeared in the cases of 5 and 10 samples, indicating that as the sample size increases, the generalization ability of MACML-IDS improves. It does not exhibit a bias toward any single attack type. The overall data distribution in Fig 9 is better than that in Fig 8, which may be due to the more distinct characteristics of attack types in the CICIDS2018 dataset compared to those in the CICIoT2023 dataset.

In summary, MACML-IDS demonstrates stable performance in few-shot scenarios. The results confirm that even with only one training sample, the model maintains a high detection rate and accuracy. As the number of training samples increases, the performance of MACML-IDS improves significantly, with no tendency to favor specific attack types.

#### 4.4.2 Results of experiment II.

In Experiment II, we evaluated the generalization capability of MACML-IDS in cross-domain scenarios by training the model on one dataset and testing it on another. The goal of this experiment is to simulate real-world situations where an intrusion detection system trained on data from one network environment needs to adapt to another network with different attack patterns.

The evaluation was conducted in two settings: (1) training on CICIDS2018 and testing on CICIoT2023, and (2) training on CICIoT2023 and testing on CICIDS2018. The results of these cross-domain evaluations are presented in Tables 8 and 9, respectively.

**Table 8:** Cross-domain detection results on CICIoT2023.

Training type	Test type	1-shot		5-shot		10-shot	
		ACC	DR	ACC	DR	ACC	DR
b,c,d	A	92.30	93.02	94.50	93.88	95.30	95.77
a,b,d	B	84.70	85.13	92.85	92.69	94.58	95.20
a,c,d	C	95.50	92.31	97.76	97.73	98.90	98.86
a,b,c	D	87.24	88.33	93.60	92.75	94.43	95.53
**Average**		89.94	89.70	94.68	94.26	95.80	96.34

**Table 9:** Cross-domain detection results on CICIDS2018.

Training type	Test type	1-shot		5-shot		10-shot	
		ACC	DR	ACC	DR	ACC	DR
A,B,C	a	98.19	97.91	99.80	99.75	100.0	100.0
A,B,D	b	94.11	93.04	98.17	96.44	100.0	100.0
A,C,D	c	97.32	96.72	99.72	97.73	99.34	99.40
B,C,D	d	99.80	99.59	100.0	100.0	100.0	100.0
**Average**		97.36	96.82	99.42	98.48	99.84	99.85

As shown in Tables 8 and 9, Table 8 presents the detection results of MACML-IDS when trained on the CICIDS2018 dataset and tested on the CICIoT2023 dataset. Table 9 presents the detection results when trained on the CICIoT2023 dataset and tested on the CICIDS2018 dataset. In both tables, a, b, c, and d represent the attack types DDoS, DoS, Brute Force, and Botnet in the CICIDS2018 dataset, respectively, while A, B, C, and D represent the attack types Mirai, Spoofing, DoS, and Recon in the CICIoT2023 dataset.

**Fig 10:**
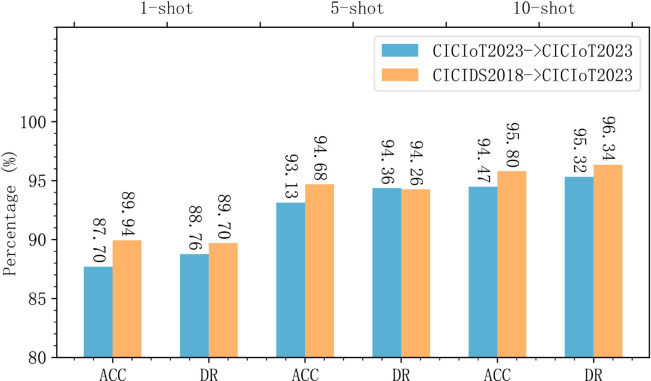
Comparison of same-domain and cross-domain experimental results on CICIoT2023.

**Fig 11:**
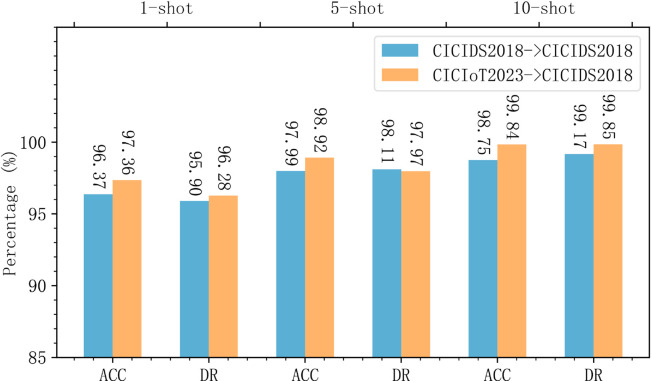
Comparison of same-domain and cross-domain experimental results on CICIDS2018.

The detection results in Tables 8 and 9 are compared with those in Figs 6 and 7. The comparison results are shown in Figs 10 and 11.

Fig 10 represents the detection results on the CICIoT2023 dataset for both same-domain and cross-domain experiments, while Fig 11 represents the detection results on the CICIDS2018 dataset. In Figs 10 and 11, in scenarios with 1, 5, and 10 training samples, MACML-IDS shows no significant decrease in detection results. We can conclude that MACML-IDS does not suffer a performance decline when trained on data from different networks, and the average accuracy and detection rate improve slightly on both the CICIoT2023 and CICIDS2018 datasets. This indicates that MACML-IDS has good generalization ability.

**Fig 12:**
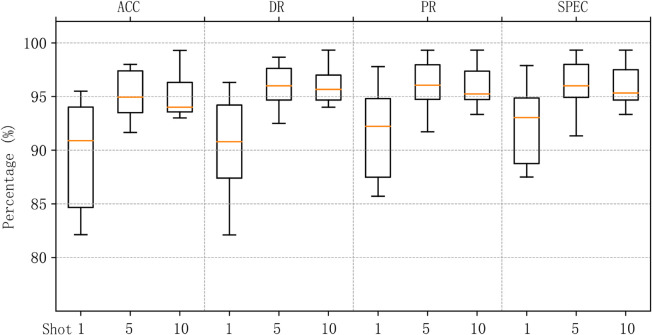
Data distribution of the cross-domain experimental results on CICIoT2023.

**Fig 13:**
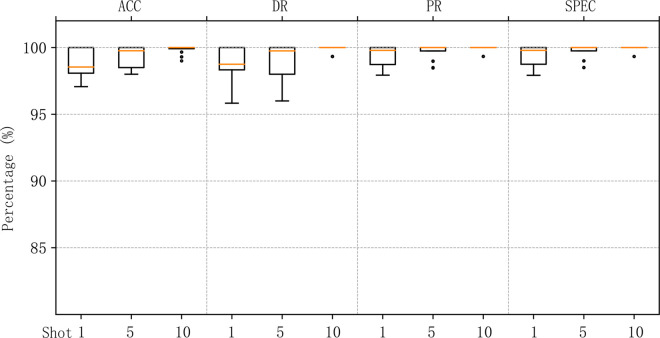
Data distribution of the cross-domain experimental results on CICIDS2018.

Figs 12 and 13 summarize the data distribution of the cross-domain experimental results for different attack types. Fig 12 shows the data distribution for the CICIoT2023 dataset, and Fig 13 shows the data distribution for the CICIDS2018 dataset. Compared to Fig 8, we observe that, in 1-shot scenario, the maximum value and median value in Fig 12 are higher, but the box plot is larger, indicating that the data distribution is more dispersed than in Fig 8. However, no outliers are observed. With the training sample size increases, the data distribution becomes more concentrated, resembling the distribution in Fig 8. Fig 13 shows a higher overall median and maximum value compared to Fig 9, and the data distribution is more concentrated. in 10-shot scenario, some outliers are observed, possibly due to one attack type having slightly worse results than the others, while the accuracy, detection rate, precision, and specificity for other attack types reach 100%.

Overall, the cross-domain experiment results confirm that MACML-IDS is capable of detecting attacks in different IoT and traditional network environments with high accuracy, making it a suitable choice for real-world intrusion detection applications where training data from target domains may be unavailable.

## 5 Discussion

### 5.1 Comparison with related work

Currently, research on few-shot IoT intrusion detection systems has made some progress. In Sect 2, we provided a brief review of related works similar to our study. Most of these studies adopt the same evaluation metrics as used in this study, allowing for direct comparison between existing methods and MACML-IDS. Given that the CICIoT2023 dataset is relatively new, we primarily perform comparisons on the more widely used CICIDS2018 dataset. The comparison mainly focuses on two aspects: the number of training samples and detection performance. Detailed comparison results are presented in Table 10.

**Table 10:** Comparison between MACML-IDS and related work.

Method	Number of Samples	ACC	DR
VAE/CVAE (2022) [39]	2800000	98.57	98.56
Graph embedding (2023) [40]	9936	99.76	97.14
Prototypical capsule network (2023) [6]	29850	95.22	96.68
IKT with NAS (2024) [31]	10000	98.75	98.85
C-FSCIL (2022) [41]	5	N/A	92.00
L2F with MAML (2022) [5]	5	96.24	97.77
L2F with MAML (2022) [5]	10	97.92	98.29
FML+FCN (2024) [42]	10	86.07	N/A
FML+ResNet (2024) [42]	10	87.27	N/A
BFS-NID (2024) [43]	5	N/A	93.17
CNNBiGRU (2024) [44]	50	97.65	97.60
BS-Agg (2025) [45]	N/A	97.90	91.70
**MACML-IDS (proposed)**	1	96.37	95.90
**MACML-IDS (proposed)**	5	97.99	98.11
**MACML-IDS (proposed)**	10	98.75	99.17

From Table 10, it can be observed that the proposed MACML-IDS consistently achieves superior detection performance compared to the other methods listed. Specifically, compared to the few-shot network intrusion detection methods such as L2F with MAML [5] and BFS-NID [43], MACML-IDS demonstrates clear advantages. In the 5-shot scenario, MACML-IDS achieves 1.75% higher accuracy and 0.34% higher detection rate than L2F with MAML, and 4.94% higher detection rate than BFS-NID. In the 10-shot scenario, MACML-IDS achieves 0.83% higher accuracy and 0.88% higher detection rate than L2F with MAML. Moreover, MACML-IDS maintains high performance even with a much lower number of training samples, outperforming several other methods, including CNNBiGRU [44], which require more samples and still do not achieve the same level of performance. This emphasizes the efficiency and effectiveness of our proposed method in achieving high detection accuracy and detection rate with fewer samples. The results demonstrate that MACML-IDS not only performs better but also offers a few-shot solution for the intrusion detection task.

### 5.2 Multiclass classification

**Fig 14:**
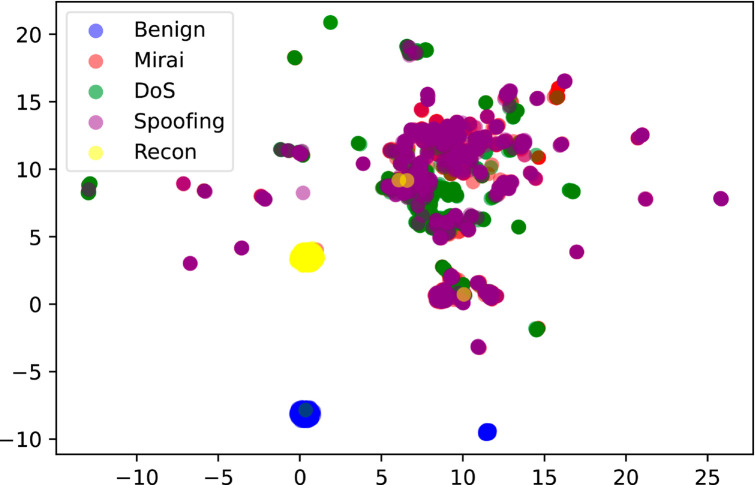
Visualization of the CICIoT2023 dataset.

**Fig 15:**
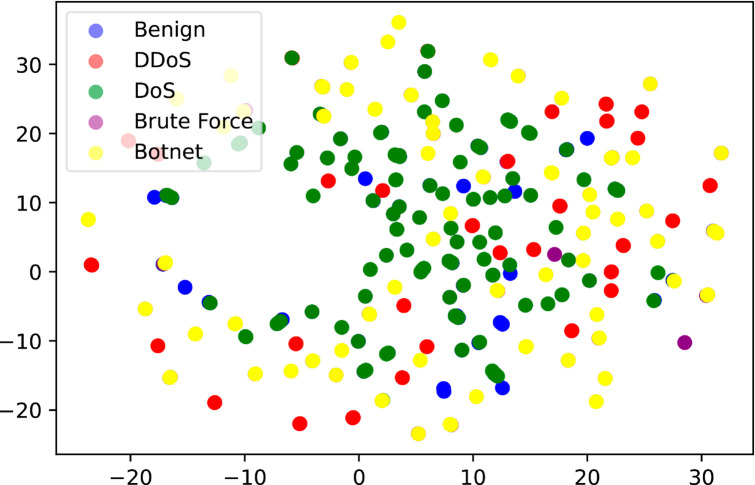
Visualization of the CICIDS2018 dataset.

In Sect 4, we performed binary classification, where MACML-IDS only distinguished between benign and attack types. To better illustrate the data distribution of various types in the preprocessed CICIoT2023 and CICIDS2018 datasets, we employed the UMAP algorithm for visualization. UMAP is a nonlinear dimensionality reduction algorithm that maps high-dimensional data to a lower-dimensional space for visualization and analysis [46]. Compared to traditional linear dimensionality reduction methods such as PCA, UMAP better preserves the nonlinear structure of the data. The visualization results of the UMAP algorithm are shown in Figs 14 and 15, where Fig 14 presents the visualization of the CICIoT2023 dataset, and Fig 15 presents the visualization of the CICIDS2018 dataset.

In Fig 14, the distribution ranges of the benign and attack types do not overlap. In Fig 15, the distribution of benign and attack types differs from that in Fig 14, but they can still be clearly distinguished, indicating that the high detection performance of MACML-IDS in binary classification on these two datasets is reasonable. However, in Fig 14, some attack types exhibit overlapping distribution ranges, suggesting that performing multiclass classification on the CICIoT2023 dataset presents significant challenges. In contrast, although the attack types in the CICIDS2018 dataset show slight overlap, they can still be distinguished, making multiclass classification on the CICIDS2018 dataset relatively less challenging.

To more comprehensively evaluate the performance of MACML-IDS, we conducted a multiclass classification task, specifically a four-class classification experiment. Since both CICIoT2023 and CICIDS2018 contain only five attack types, and some attack types overlap, we selected four attack types from each dataset for fairness and comparability. We used the DDoS, Brute Force, Botnet, and Benign classes from the CICIDS2018 dataset as the training set and the Mirai, DoS, Spoofing, and Benign classes from the CICIoT2023 dataset as the test set.

**Fig 16:**
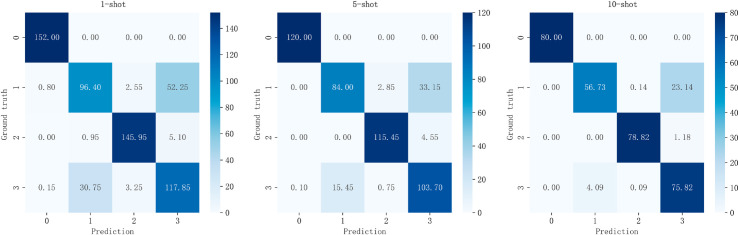
Heatmaps of average confusion matrices in 1, 5 and 10-shot scenarios.

Fig 16 presents the heatmaps of the average confusion matrices obtained by MACML-IDS in 1, 5 and 10-shot scenarios. Specifically, we trained MACML-IDS on the CICIDS2018 dataset usin 1, 5 and 10-shot scenarios, then tested it multiple times on the CICIoT2023 dataset. The confusion matrices from these tests were averaged to produce the data in Fig 16 to reduce errors.

**Fig 17:**
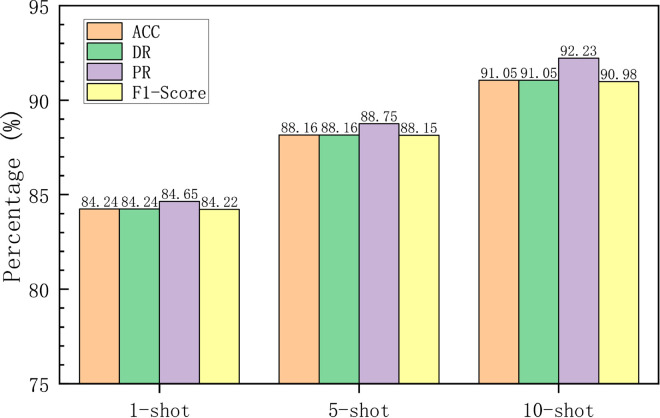
Multiclass detection results in 1, 5 and 10-shot scenarios.

In Fig 16, the vertical axis represents the labels of the Mirai, DoS, Spoofing, and Benign types from the CICIoT2023 dataset, while the horizontal axis represents the predicted labels by MACML-IDS. The main diagonal represents correctly classified samples, while other areas indicate misclassifications. The deeper the color along the main diagonal, the better the model’s detection performance. Fig 16 shows that MACML-IDS performs well in recognizing Mirai, Spoofing, and Benign samples, but its recognition ability for DoS is relatively weak. A detailed analysis is provided in Fig 17.

Fig 17 presents four metrics: accuracy, detection rate, precision, and F1-score. The results indicate that, in 1-shot scenario, MACML-IDS achieves an average accuracy of 84.24% and an F1-score of 84.22. in 10-shot scenario, the average accuracy reaches 91.05% and the F1-score reaches 90.98. These results suggest that although performing multiclass classification on the CICIoT2023 dataset poses significant challenges, MACML-IDS still exhibits good detection performance.

## 6 Conclusion

In this paper, we propose a marrying attention and convolution-based meta-learning (MACML) method for few-shot IoT intrusion detection to address the limitations of traditional intrusion detection methods in low-data environments. MACML integrates the advantages of self-attention mechanisms and convolutional neural networks. The self-attention mechanism is employed to capture global dependencies between network flows, while the CNN is utilized to extract local features, compensating for the self-attention mechanism’s limited focus on local information. Additionally, MACML adopts an optimization-based meta-learning framework, enabling the model to rapidly acquire prior knowledge from a limited number of training samples and generalize to new tasks, thereby enhancing the adaptability and detection performance of the intrusion detection system.

Extensive experiments were conducted on the CICIDS2018 and CICIoT2023 datasets to validate the effectiveness of MACML-IDS. The experimental results demonstrate that with only 10 training samples, MACML-IDS achieves an average accuracy of 98.75% and an average detection rate of 99.17% on the CICIDS2018 dataset, while attaining an average accuracy of 94.47% and an average detection rate of 95.32% on the CICIoT2023 dataset. Compared to conventional deep learning-based intrusion detection methods, MACML-IDS exhibits superior detection capability and generalization performance in few-shot scenarios. Notably, in cross-domain detection experiments, MACML-IDS maintains stable detection performance, demonstrating its robustness. Furthermore, in multiclass classification tasks, MACML-IDS continues to show strong classification performance, indicating its broad applicability in IoT intrusion detection.
